# The Evidence-Practice Gap in Testosterone Therapy for Prostate Cancer: A Narrative Review

**DOI:** 10.7759/cureus.110973

**Published:** 2026-06-16

**Authors:** Mohammed Aly, Anisha Joseph, Tarik Amer

**Affiliations:** 1 Urology, Forth Valley Royal Hospital, Glasgow, GBR; 2 Internal Medicine, National Health Service (NHS) Grampian, Aberdeen, GBR; 3 Urology, National Health Service (NHS) Fife, Kircaldy, GBR

**Keywords:** active surveillance, evidence-based practice, hypogonadism, prostate cancer, shared decision-making, survivorship, testosterone replacement therapy (trt)

## Abstract

The contraindication to testosterone replacement therapy (TRT) in men with prostate cancer (PCa) has been progressively challenged by contemporary evidence. Over recent years, increasing data suggest that TRT does not increase biochemical recurrence, disease progression, or PCa-specific mortality in carefully selected men, including those managed by active surveillance and those treated with radical prostatectomy or radiotherapy. Despite this convergence in the evidence, clinical practice has lagged. Major society guidelines continue to describe the available data as inadequate; many clinicians decline TRT in any man with a history of PCa, and symptomatic hypogonadism in survivors remains undertreated. This narrative review synthesises the contemporary evidence and characterises the persistent gap between evidence and clinical practice. It examines the principal drivers of that gap, which include conservative interpretation of guideline language, medico-legal anxiety, concerns surrounding on-treatment biochemical monitoring, the absence of randomised trials in this specific population, multidisciplinary fragmentation, and limited exposure during training. A practical clinical framework for shared decision-making and on-treatment surveillance, relevant to the UK and European practice, is proposed. The review concludes that an absolute contraindication is no longer tenable in men with symptomatic hypogonadism and a history of treated PCa, and that this population deserves a contemporary, evidence-informed conversation about TRT.

## Introduction and background

Survivorship is now a major part of prostate cancer (PCa) care. Five-year cancer-specific survival for localised disease now exceeds 95%, and the population of men living with a history of PCa is large, growing, and ageing. The key questions are increasingly about quality of life rather than survival alone. Within that domain, sexual function, fatigue, bone health, mood, and metabolic status are among the most strongly cited determinants of post-treatment quality of life [1,2].

Hypogonadism, the clinical syndrome of testosterone deficiency, is defined by persistently low serum testosterone together with characteristic symptoms. It is useful to distinguish biochemical hypogonadism, which denotes a low measured testosterone alone, from symptomatic hypogonadism, in which the biochemical deficiency is accompanied by clinical features such as reduced libido, fatigue, low mood, and loss of muscle mass. Symptomatic testosterone deficiency is common in this population, affecting 20-35% of men following radical prostatectomy and 35-50% of men following radiotherapy, with prevalence rising further in those exposed to androgen deprivation therapy (ADT), the therapeutic suppression of testosterone used to treat PCa [3]. Against this background, testosterone replacement therapy (TRT) might seem a natural part of survivorship care, but it has not been routinely used in this setting. The reason is largely historical: because PCa was understood to be an androgen-driven disease, restoring testosterone was long assumed to risk reactivating it. This relationship has been governed for the better part of a century by an absolute contraindication founded in the seminal 1941 work of Huggins and Hodges [4], a position that has been progressively challenged as the underlying biology has become better understood. Although understanding of PCa biology has evolved since the late 2000s, particularly following the saturation model proposed by Morgentaler and Traish [5], clinical practice has been slow to follow.

In 2025 and into 2026, several reviews reached similar conclusions. Multiple systematic and scoping reviews have appeared within months of one another [6-9], all reaching a similar conclusion: across active surveillance (the structured monitoring of low-risk disease without immediate treatment), post-radical prostatectomy, and post-radiotherapy populations, TRT in carefully selected men is not associated with increased biochemical recurrence (a rise in prostate-specific antigen (PSA) signalling possible disease activity), disease progression, metastasis, or cancer-specific mortality during available follow-up. The British Society for Sexual Medicine (BSSM) has issued a UK consensus statement endorsing the cautious use of TRT in PCa survivors [8]. The evidence has moved faster than clinical practice. This review synthesises the contemporary evidence, characterises the persistent evidence-practice gap, explores its drivers, proposes a practical framework for assessment and monitoring relevant to the UK and European clinical contexts, and identifies the structural changes that would help to close the gap.

## Review

Methodology

This article is a narrative review of the literature. PubMed, EMBASE, and the Cochrane Library were searched from inception to January 2026 using combinations of the terms “testosterone,” “testosterone replacement therapy,” “androgen,” “hypogonadism,” “prostate cancer,” “active surveillance,” “radical prostatectomy,” “radiotherapy,” and “biochemical recurrence,” with no language restriction applied at the search stage. This was supplemented by hand-searching of the reference lists of retrieved articles and of relevant society guidelines published in the United Kingdom, Europe, and North America. Studies were considered eligible if they reported oncological or biochemical outcomes in hypogonadal men receiving TRT during active surveillance or following definitive treatment for PCa; foundational mechanistic studies and contemporary society guidelines were additionally included to provide biological and clinical context. Conference abstracts without full data and, at the screening stage, non-English-language articles were not prioritised. Consistent with a narrative rather than systematic design, articles were identified and selected by the authors by consensus rather than through a formal dual-reviewer screening process, and neither a quantitative meta-analysis nor a formal risk-of-bias scoring exercise was undertaken; accordingly, a Preferred Reporting Items for Systematic Reviews and Meta-Analyses-style study selection diagram was not appropriate. Because the great majority of the available evidence is observational, the potential for selection bias, residual confounding, and limited follow-up was weighed qualitatively when interpreting individual studies, and these limitations are made explicit throughout.

The origin of a dogma

The contraindication to TRT in men with PCa originates almost entirely from a single paper. In 1941, Huggins and Hodges reported tumour regression following castration and biochemical worsening following exogenous testosterone administration in patients with metastatic PCa [4]. The work was foundational; it earned Huggins a Nobel Prize and established ADT as a cornerstone of advanced PCa management. Its inferential reach, however, was modest. The androgen administration arm of the study rested on three patients monitored with prostatic acid phosphatase assays that bear little resemblance to contemporary biomarkers. There were no controls, no blinding, no defined endpoints in any modern sense, and no formal statistical analysis. The conclusion, which became dogma, was the simplest possible inference: testosterone is fuel for PCa; therefore restoration of testosterone in any man with any history of PCa is dangerous.

The 1941 observations were extrapolated far beyond their evidentiary base. The patients in question had advanced, metastatic, hormone-naive disease, with the high circulating androgen levels of untreated late-stage cancer. The men in whom TRT might today be considered have low- or intermediate-risk localised disease, frequently several years past curative treatment, with undetectable or stable PSA. The biological situations are not equivalent. Yet, for several decades, there were no better data, and clinical inertia, supported by guideline language and reinforced by medico-legal anxiety, sustained the contraindication well into the twenty-first century. Routine teaching in urology, general medicine, and primary care continued to characterise testosterone as universally unsafe in any man with a history of PCa, with little distinction between disease states.

The first serious challenge came in 2009 with the saturation model proposed by Morgentaler and Traish, which posited that prostatic androgen receptors are saturated at relatively low circulating testosterone concentrations, in the region of 250 ng/dL (8.7 nmol/L) [5]. The model drew on receptor binding kinetics, observations of intraprostatic androgen levels, and an emerging body of clinical observation. Under this model, restoring testosterone from hypogonadal to physiological levels does not measurably increase androgenic stimulation of the prostate beyond a low threshold, because receptors are already maximally engaged. The model was biologically coherent and explained an otherwise paradoxical pattern in the clinical literature: the well-established efficacy of androgen deprivation at castrate testosterone levels alongside the apparent safety of testosterone supplementation in eugonadal men. It also predicted the empirical observation, subsequently confirmed across multiple cohorts, that PSA does not generally rise meaningfully when hypogonadal men receive TRT.

Empirical support for the saturation hypothesis emerged from the multicentre cohort reported by Khera and colleagues, who followed hypogonadal men on TRT over 12 months and documented mean PSA changes of less than 0.5 ng/mL during therapy [10]. Subgroup analysis confirmed that men with the lowest baseline testosterone, in whom the largest receptor activation effect would be expected, exhibited the largest PSA rises, while men with near-physiological baseline values showed essentially flat trajectories. These findings were consistent with the predictions of the saturation model and inconsistent with a linear stimulatory effect of testosterone on prostate tissue.

The saturation model has not gone unchallenged. Kim has argued that the experimental basis for receptor saturation thresholds was extrapolated beyond the limitations of the original studies, that the dose-response data underpinning the model were reconstructed on questionable axes, and that the model insufficiently accounts for tumour heterogeneity and the dynamic nature of androgen receptor signalling in malignant tissue [11]. In response, Morgentaler and Traish have maintained that more than a decade of accumulating clinical data is consistent with receptor saturation as an accurate framework, rather than a mere hypothesis [12]. For the purposes of the present review, the saturation model should not be read as proof that TRT is universally safe in any setting; rather, it offers a biologically coherent framework for the empirical observations of contemporary practice, and a counterweight to the linear assumption of androgen stimulation that has driven the historical contraindication. The clinical question is no longer whether physiological-range testosterone is uniformly dangerous in men with a history of PCa, but in whom, when, and under what surveillance arrangements it may be reasonable to consider.

The changing evidence

A substantial body of contemporary data, accumulated over the last 15 years, has progressively weakened the case for an absolute contraindication. The evidence may be grouped under five complementary headings: population-level and randomised data, studies in men managed by active surveillance, studies in men following radical prostatectomy, studies in men following radiotherapy, and contemporary evidence syntheses.

Population-Level and Randomised Evidence

The Endogenous Hormones and Prostate Cancer Collaborative Group analysis of 18 prospective studies, encompassing thousands of men with measured serum androgens, demonstrated no significant association between circulating endogenous androgens and PCa risk [13]. This pooled analysis remains one of the most comprehensive examinations of the relationship between physiological testosterone exposure and PCa incidence and challenges the simple linear model that underlies the historical contraindication.

Observational data have provided additional reassurance. The Swedish nationwide registry analysis by Loeb and colleagues, including 38,570 men with PCa and 192,838 matched controls, found no increased PCa risk associated with TRT exposure, and a lower risk of aggressive disease in TRT users (odds ratio (OR = 0.50, 95% confidence interval (CI) = 0.37-0.67) [14]. The signal was particularly notable for high-risk disease and persisted across sensitivity analyses, suggesting that the inverse association may not be fully explained by confounding. Although registry data cannot exclude residual confounding from healthier patient selection in those receiving TRT, the size and consistency of the signal across endpoints argue against TRT exposure conferring meaningful oncological harm at the population level.

Randomised data emerged from the TRAVERSE programme, a placebo-controlled trial of TRT in middle-aged and older men at increased cardiovascular risk [15]. The prostate-safety substudy, prospectively designed and adequately powered, demonstrated no significant difference in the incidence of high-grade PCa or in any PCa between TRT and placebo arms during a median follow-up of 33 months [16]. While the trial did not enrol men with established PCa, the population was selected for the very comorbidity profile in which TRT prescribing decisions are most frequently considered, and the absence of any signal toward increased PCa events provides important reassurance about exogenous testosterone at physiological doses in a moderate-risk older male population.

A 2026 systematic review and meta-analysis by Garcia-Becerra and colleagues, encompassing 41 randomised controlled trials with a pooled population of 11,161 men, examined cardiovascular and PCa endpoints together [17]. The analysis found no statistically significant association between TRT and major adverse cardiovascular events (OR = 0.83, 95% CI = 0.52-1.32), PCa events (OR = 0.88, 95% CI = 0.52-1.51), or clinically significant PCa events (OR = 1.13, 95% CI = 0.39-3.26). The wide CIs, most marked for clinically significant PCa, reflect relatively few events and substantial heterogeneity between the pooled trials in testosterone formulation, dose, treatment duration, and baseline population; the pooled estimates are therefore best interpreted as showing no signal of harm rather than as positive proof of safety. The breadth of this synthesis, drawing on trials of varying duration and patient population, nonetheless provides pooled randomised reassurance that was not previously available. Together, the population, observational, and randomised evidence indicates that exposure to physiological-range testosterone in hypogonadal men does not generate a measurable increase in PCa risk.

Active Surveillance

Several cohorts have specifically examined TRT in men managed by active surveillance for low- and favourable intermediate-risk PCa, a population in whom symptomatic hypogonadism is increasingly recognised but in whom historical practice has reflexively withheld therapy.

In the controlled biopsy comparison reported by Kacker and colleagues, men on active surveillance receiving TRT underwent the same scheduled biopsy protocol as controls [18]. Gleason upgrading occurred in 10.7% of TRT-treated men compared with 9.4% of controls (p = 0.732). Although the cohort was modest in size, the matched biopsy methodology represents one of the few attempts to disentangle disease progression from detection bias, a recurrent confounder in observational TRT literature. The propensity-matched analysis by Daza and colleagues, drawing on contemporary surveillance cohorts, found no significant difference in treatment-free survival between TRT-treated and untreated men, with follow-up sufficient to capture early progression events [19]. The largest analysis to date is the SEER-Medicare population study by Kaplan-Marans and colleagues, in which 167 TRT-treated men were compared with 6,658 controls drawn from a contemporaneous national surveillance population [20]. The TRT-exposed cohort demonstrated a lower observed treatment conversion rate (16.8% versus 21.9%; adjusted hazard ratio (HR) = 0.66, 95% CI = 0.46-0.97; p = 0.033). While this signal must be interpreted cautiously given the potential for residual healthier-patient confounding, the absence of harm at this scale is reassuring and inconsistent with the assumption that TRT accelerates disease behaviour in surveillance populations.

The contemporary cohort reported by Applewhite and colleagues, with a mean follow-up of 6.1 years, recorded no metastases and no PCa-specific deaths among 43 TRT-treated patients on active surveillance, despite the inclusion of men with biopsy-confirmed Gleason grade group 1 and selected grade group 2 disease [21]. The duration of follow-up in this series is particularly relevant: many earlier cohorts were criticised for short follow-up that could obscure late progression events, and the Applewhite data extend confident observation into the timeframe in which the natural history of unfavourable disease typically manifests.

Post-radical Prostatectomy

Evidence in men who have undergone radical prostatectomy is among the strongest available because of the simplicity of biochemical monitoring after surgical removal of the prostate. In the absence of residual benign or malignant prostatic tissue, any persistent or rising serum PSA represents disease activity, and the signal-to-noise ratio of postoperative PSA surveillance is favourable for detecting recurrence.

Khera and colleagues reported on hypogonadal men receiving TRT after radical prostatectomy with no biochemical recurrences attributable to TRT, and stable PSA on treatment [22]. The Pastuszak series of 103 men, including 26 with high-risk pathology at prostatectomy, recorded no metastatic events and stable PSA across a mean follow-up of 27 months [23]. Sarkar and colleagues, drawing on SEER-Medicare data linking PCa registry information to TRT prescription records, examined men following both radical prostatectomy and radiotherapy and found that TRT exposure after definitive treatment was not associated with increased risks of biochemical recurrence, all-cause mortality, or PCa-specific mortality [24]. The analysis adjusted for multiple potential confounders including age, comorbidity, treatment modality, and PSA at TRT initiation.

Ahlering and colleagues, in a single-institution comparative cohort, reported a paradoxical but consistent observation: biochemical recurrence after radical prostatectomy was lower in men subsequently treated with TRT compared with untreated matched controls [25]. The authors proposed several biologically plausible mechanisms, including the differential effect of testosterone on benign versus malignant tissue and the possibility that physiological androgen replenishment supports tissue repair processes that limit microscopic residual disease. Whatever the mechanism, the direction of effect runs against the historical assumption of androgen-driven recurrence and adds to the accumulating body of evidence that TRT is not oncologically detrimental in this setting.

The most contemporary and largest single-institution analysis comes from Flores and colleagues at Memorial Sloan Kettering Cancer Center, who reported on 198 TRT-treated men compared with 5,001 untreated controls following radical prostatectomy for grade group 1-3 organ-confined disease [26]. The analysis demonstrated a non-significantly decreased rate of biochemical recurrence with TRT (HR = 0.84, 95% CI = 0.48-1.46) over a substantial follow-up. The inclusion of patients with grade group 3 disease, traditionally regarded as intermediate-to-high-risk pathology, is particularly relevant; the absence of an adverse signal in this subgroup supports a careful broadening of the candidate population beyond the very low-risk cases that earlier series exclusively considered.

Post-radiotherapy

Evidence in men following radiotherapy is more limited but consistent in direction. The Sarosdy series of 31 men receiving TRT after brachytherapy for early PCa reported no biochemical recurrences during a median follow-up of five years, despite the inclusion of men with both low and selected intermediate-risk disease [27]. The interpretation of post-radiation PSA is complicated by the persistence of residual prostatic tissue and the well-described phenomenon of PSA bounce, and a robust monitoring strategy requires familiarity with these phenomena. Nonetheless, the absence of biochemical recurrence in this cohort across a clinically relevant follow-up period was reassuring at the time and has been corroborated in subsequent series.

Pastuszak and colleagues reported a multi-institutional series of 98 men receiving TRT after radiation therapy, spanning low-, intermediate-, and high-risk pre-treatment disease, with a mean follow-up of approximately 41 months [28]. The cohort included men treated with external beam radiotherapy, brachytherapy, and combination modalities. Biochemical recurrence rates were low and not significantly elevated compared with published baselines for matched populations. The Balbontin cohort, in which 38 men with hypogonadism after brachytherapy received long-acting intramuscular testosterone undecanoate, similarly demonstrated reassuring oncological outcomes during prospective monitoring [29]. Although the radiotherapy literature lacks the granularity of the post-prostatectomy data, the absence of an adverse signal across cohorts and treatment modalities supports a measured approach to TRT in this population, with attention to baseline imaging and post-radiation PSA trajectory.

Synthesis from contemporary reviews

Within the last 12 months, four major evidence syntheses have examined TRT in men with PCa, each with a distinct methodological orientation. The Gibson scoping review covered 12 studies in post-treatment populations and found no signal of increased biochemical recurrence or progression [6]. The Santucci systematic review in BJU International examined 19 studies across active surveillance, post-radical prostatectomy, and post-radiotherapy populations, reporting biochemical recurrence rates of 0-7% post-prostatectomy and 0-6% post-radiotherapy, with no signal of increased risk attributable to TRT [7]. A further systematic review by Robinson and colleagues, focused on a slightly different inclusion frame, reached convergent conclusions [9]. The BSSM consensus statement of 2026 brings these strands together into a UK-relevant clinical position, supporting the cautious use of TRT in well-selected PCa survivors with symptomatic secondary hypogonadism, providing structured surveillance and explicit shared decision-making [8].

The signal across these data is consistent. Across study designs, treatment modalities, and follow-up durations, TRT in carefully selected men with treated or surveilled PCa has not been associated with adverse oncological outcomes. Despite this consistency, the available evidence carries important methodological limitations. Most studies are observational and subject to selection bias, as healthier men with more favourable disease are more likely to be offered TRT; retrospective cohorts are additionally vulnerable to residual confounding and to immortal time bias, in which the interval preceding treatment initiation can artefactually favour treated patients. The consistent absence of an adverse signal should therefore be interpreted as an absence of evidence of harm rather than as definitive evidence of safety. Randomised data in men with treated PCa are lacking, long-term follow-up is limited, and evidence remains sparse in several specific groups, including high-risk and node-positive disease, men with a persistently detectable PSA, and those previously exposed to prolonged ADT. In practice, this supports careful patient selection and structured monitoring rather than a uniform approach (Table 1).

**Table 1:** Summary of representative studies of testosterone replacement therapy in men with prostate cancer, grouped by clinical setting. A dash indicates not applicable or not reported. AS = active surveillance; BCR = biochemical recurrence; csPCa = clinically significant prostate cancer; CV = cardiovascular; EH&PCa = Endogenous Hormones and Prostate Cancer Collaborative Group; GG = grade group; HR = hazard ratio; N = number of participants (TRT group/control group where applicable); OR = odds ratio; PCa = prostate cancer; PSA = prostate-specific antigen; RCT = randomised controlled trial; RP = radical prostatectomy; RT = radiotherapy; TRT = testosterone replacement therapy.

Study	Design	Population	N	Follow-up	Key oncological outcome
Population-level and randomised evidence					
EH&PCa Collaborative Group, 2008 [13]	Pooled analysis (18 prospective studies)	Men with measured serum androgens	—	Varied	No association between endogenous androgens and PCa risk
Loeb et al., 2017 [14]	National registry, matched	PCa cases and matched controls	38,570 vs 192,838	Up to ~5 years	No increased risk; lower aggressive disease in TRT users (OR = 0.50)
TRAVERSE (Lincoff et al./Bhasin et al.), 2023 [15,16]	RCT, placebo-controlled (prostate substudy)	Older men at raised CV risk (no established PCa)	~5,200	Median: 33 months	No difference in high-grade or any PCa vs. placebo
Garcia-Becerra et al., 2026 [17]	Systematic review and meta-analysis (41 RCTs)	Hypogonadal men	11,161	Varied	No association with PCa events (OR = 0.88) or csPCa (OR = 1.13)
Active surveillance					
Kacker et al., 2016 [18]	Controlled biopsy comparison	AS, low/favourable intermediate-risk	Modest	Scheduled biopsy	Gleason upgrading 10.7% (TRT) vs 9.4% (control), p = 0.732
Daza et al., 2023 [19]	Propensity-matched cohort	AS	Matched cohorts	Sufficient for early events	No difference in treatment-free survival
Kaplan-Marans et al., 2024 [20]	Population-based (SEER-Medicare)	AS	167/6,658	Population follow-up	Lower treatment conversion (16.8% vs 21.9%; HR = 0.66)
Applewhite et al., 2025 [21]	Contemporary cohort	AS, GG1 and selected GG2	43	Mean: 6.1 years	No metastases; no PCa-specific deaths
Post-radical prostatectomy					
Khera et al., 2009 [22]	Cohort	Hypogonadal men post-RP	Series	On-treatment	No BCR attributable to TRT; stable PSA
Pastuszak et al., 2013 [23]	Cohort (including 26 high-risk)	Post-RP	103	Mean: 27 months	No metastatic events; stable PSA
Sarkar et al., 2020 [24]	Population-based (SEER-Medicare)	Post-RP and post-RT	Large	Registry follow-up	No increased BCR, all-cause or PCa-specific mortality
Ahlering et al., 2020 [25]	Comparative cohort	Post-RP	Matched cohorts	Comparative	Lower BCR with TRT vs. controls
Flores et al., 2025 [26]	Single-institution comparative	Post-RP, GG1-3 organ-confined	198/5,001	Substantial	Non-significantly decreased BCR (HR = 0.84)
Post-radiotherapy					
Sarosdy, 2007 [27]	Cohort	Post-brachytherapy, low/selected intermediate-risk	31	Median: 5 years	No biochemical recurrences
Pastuszak et al., 2015 [28]	Multi-institutional cohort	Post-RT, low/intermediate/high-risk	98	Mean: ~41 months	Low BCR; not significantly elevated
Balbontin et al., 2014 [29]	Prospective cohort	Post-brachytherapy	38	Prospective	Reassuring oncological outcomes
Contemporary evidence syntheses					
Gibson et al., 2025 [6]	Scoping review (12 studies)	Post-treatment populations	—	—	No signal of increased BCR or progression
Santucci et al., 2025 [7]	Systematic review (19 studies)	AS/post-RP/post-RT	—	—	BCR 0-7% (post-RP), 0-6% (post-RT); no increased risk
Robinson et al., 2025 [9]	Systematic review	Treated PCa	—	—	Convergent: TRT not contraindicated after definitive treatment

A practical framework for assessment and monitoring

Closing the evidence-practice gap requires more than acknowledgement that the evidence has moved. It requires a defined clinical pathway that allows individual clinicians to act on the evidence without venturing into improvised territory. The following framework, drawn from the BSSM 2026 consensus; the American Urological Association, Canadian Urological Association, and European Association of Urology guidelines [8,30-32]; and the practical realities of NHS outpatient practice, summarises an approach to candidate selection, pre-treatment assessment, and on-treatment monitoring. It should be read as a pragmatic synthesis informed by current guidelines and expert opinion rather than as a set of formally evidence-graded recommendations. The on-treatment monitoring schedule is summarised in Table 2.

**Table 2:** Proposed on-treatment monitoring schedule for testosterone replacement therapy in men with a history of prostate cancer. MDT = multidisciplinary team; MRI = magnetic resonance imaging; PSA = prostate-specific antigen

Parameter	Schedule	Target or action threshold
Total testosterone	Baseline, 6 weeks, then 6-monthly	Maintain mid-physiological range (15-25 nmol/L; 450-700 ng/dL); avoid supraphysiological levels
PSA	Baseline, then 3, 6, 9 and 12 months in year 1; 6-monthly thereafter once stable	Investigate a velocity >0.5 ng/mL per year, an absolute rise >1.0 ng/mL above baseline, or any breach of a pre-specified ceiling; refer to MDT
Haematocrit	Baseline, 6 weeks, then 6-monthly	Dose reduction or discontinuation if sustained >54%
Digital rectal examination	Annually	Investigate any new palpable abnormality
Multiparametric MRI	Not routine; as clinically indicated	Consider for concerning PSA change, palpable abnormality, or new local symptoms
Symptom and cardiovascular assessment	Each review	Reassess clinical benefit; manage cardiovascular risk

Candidate Selection

Candidate selection should be driven by symptomatic hypogonadism rather than serum testosterone alone. Biochemical confirmation requires two early-morning total testosterone measurements below the laboratory reference range, ideally accompanied by a free or calculated free testosterone estimation, sex hormone-binding globulin, and luteinising hormone to support the diagnosis of primary or secondary hypogonadism. Symptoms most likely to respond to TRT include reduced libido, erectile dysfunction not solely explained by post-treatment anatomical change, fatigue, low mood, and loss of lean body mass. Where any of these symptoms is the dominant clinical concern, a TRT conversation is reasonable provided the PCa history is well-characterised.

Disease characterisation determines candidacy. Men with biochemically controlled disease following radical prostatectomy, with stable undetectable PSA for at least 12 months, are the strongest candidates. Men managed on active surveillance for grade group 1 or selected favourable grade group 2 disease, with stable PSA and stable imaging, are reasonable candidates after multidisciplinary discussion. Men following definitive radiotherapy with a stable post-treatment PSA nadir and no PSA bounce within the preceding 12 months may be considered, with the recognition that radiotherapy follow-up requires familiarity with post-radiation PSA dynamics. Men with biochemical recurrence, evidence of residual disease, or ongoing oncological treatment should not generally be offered TRT outside a research setting.

Pre-treatment Assessment

Pre-treatment assessment should include full blood count with haematocrit, a current lipid profile, fasting glucose or HbA1c, digital rectal examination, and a documented PSA trend over at least six months. For men on active surveillance, a baseline or recent multiparametric MRI provides a useful reference for subsequent monitoring. Bone mineral density assessment is reasonable in men with prolonged prior ADT exposure, both as part of survivorship care and because bone health is among the metabolic dividends of effective TRT. The off-label nature of the prescription should be documented explicitly in the clinical record, alongside a clear summary of the discussion.

On-Treatment Monitoring

On-treatment monitoring should be structured and proportionate. PSA should be measured at baseline, three-monthly during the first year (at 3, 6, 9, and 12 months), and six-monthly thereafter once stability has been confirmed. The expected behaviour of PSA on TRT is a modest rise of approximately 0.1-0.5 ng/mL within the first three months, reflecting the normalisation of physiological serum testosterone rather than disease activity. This rise typically plateaus and does not progress. A PSA velocity exceeding 0.5 ng/mL per year, an absolute rise of more than 1.0 ng/mL above baseline, or any breach of a pre-specified safety ceiling (for example, return to a value above the post-treatment nadir threshold for biochemical recurrence) warrants pause, repeat measurement, and discussion at the uro-oncology multidisciplinary team meeting.

Serum testosterone should be measured at six weeks and at six-monthly intervals on treatment, with the goal of maintaining total testosterone in the mid-physiological range (approximately 15-25 nmol/L or 450-700 ng/dL). Higher concentrations confer no additional symptomatic benefit and unnecessarily increase the haematocrit, lipid, and theoretical prostatic exposure risks. Haematocrit must be checked at baseline, at six weeks, and at six-monthly intervals thereafter; sustained values above 54% mandate dose reduction or discontinuation. An annual digital rectal examination remains appropriate in all men on TRT with a history of PCa. Multiparametric MRI is not routinely indicated but should be considered where there is a concerning change in PSA, palpable abnormality on examination, or new clinical symptoms suggestive of local recurrence.

Discontinuation and Shared Care

**Figure 1:**
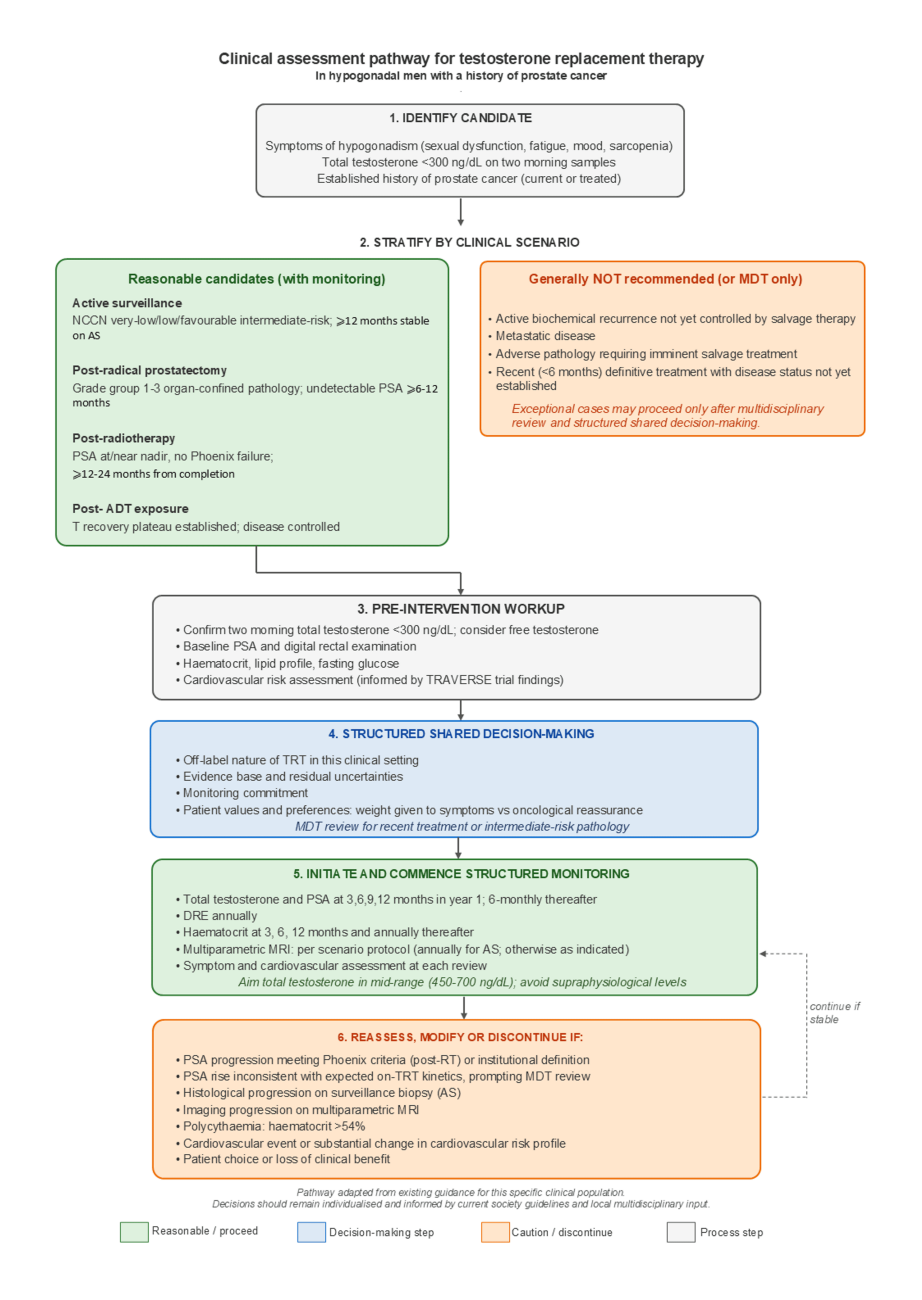
Clinical assessment pathway for testosterone replacement therapy in hypogonadal men with a history of prostate cancer. The pathway outlines six steps: candidate identification, clinical stratification by treatment setting and disease characteristics, pre-TRT workup, structured shared decision-making, treatment initiation with structured monitoring, and criteria for reassessment or discontinuation. The dashed arrow indicates iterative on-treatment review. Management should be individualised and guided by current society guidelines and multidisciplinary input. ADT = androgen deprivation therapy; AS = active surveillance; MDT = multidisciplinary team; mpMRI = multiparametric magnetic resonance imaging; NCCN = National Comprehensive Cancer Network; PSA = prostate-specific antigen; RP = radical prostatectomy; RT = radiotherapy; TRT = testosterone replacement therapy; TRAVERSE = Testosterone Replacement Therapy for Assessment of Long-Term Vascular Events and Efficacy Response in Hypogonadal Men. The figure was created by the authors using Microsoft PowerPoint (Microsoft Corporation). No generative AI tools were used.

Triggers for discontinuation include confirmed biochemical recurrence, radiological or histological evidence of disease progression, sustained haematocrit elevation, the emergence of new contraindications such as severe untreated obstructive sleep apnoea, or patient preference. Shared care arrangements with primary care, with annual specialist review of stable patients, are appropriate and reflect the realities of NHS capacity. Less stable patients should remain under direct urological or andrological follow-up. At all stages, the clinical record should reflect the off-label nature of the prescription, the surveillance plan, and the patient’s understanding of the residual uncertainty. A pragmatic clinical pathway operationalising these principles is summarised in Figure 1.

The persistent practice gap

Despite this, prescribing practice remains cautious. Three observations together describe the current state of the gap.

First, major guideline statements continue to characterise the evidence as inadequate. The American Urological Association 2018 testosterone deficiency guideline, which remains the most widely cited statement of its kind in English-language practice, advises that men with a history of PCa should be counselled on the “inadequate evidence to quantify the risk-benefit ratio of testosterone therapy” [30]. This language has not been substantively revised in the intervening years and predates the most informative contemporary studies, including the TRAVERSE prostate substudy [16], the SEER-Medicare active surveillance analysis [20], the Memorial Sloan Kettering post-prostatectomy cohort [26], and the 2025-2026 systematic reviews [6,7,9,17]. Conservative interpretation by clinicians of guideline language framed in the negative inevitably tilts decision-making toward declining TRT in any man with a PCa history, even where the evidence increasingly supports a more nuanced approach.

Second, prescribing surveys reveal substantial practitioner reluctance. Although the Canadian Urological Association guideline acknowledges that TRT may be considered in selected low-risk PCa survivors [31], the European Association of Urology likewise permits cautious testosterone therapy in symptomatic men surgically treated for localised PCa who are without evidence of active disease, restricting treatment to those at low risk of recurrence with a stable, suppressed PSA [32], and the BSSM 2026 consensus extends this position for UK practice [8]. Despite these converging guideline positions, real-world prescribing remains low. Patients with any history of PCa are routinely declined TRT in primary and secondary care alike, frequently without explicit reference to disease characteristics or current PSA stability. The BSSM consensus statement explicitly identifies this gap as a primary motivation for its publication, observing that symptomatic hypogonadal men with treated PCa represent one of the largest groups of patients in whom an evidence-based intervention is consistently withheld.

Third, hypogonadism in PCa survivors is undertreated as a clinical problem in its own right. The prevalence of symptomatic hypogonadism after radical prostatectomy or radiotherapy is high [3], and its functional and quality-of-life consequences (sexual dysfunction, fatigue, depression, sarcopenia, and cardiometabolic decline) are well documented and consistently under-addressed in survivorship pathways. The combination of common and consequential hypogonadism with a residual blanket contraindication to TRT means that a substantial cohort of survivors continues to live with treatable symptoms. The mismatch between the evidence and the practice in 2026 is therefore not merely an academic concern. It has direct consequences for symptomatic men and represents an avoidable harm of clinical inertia.

Why the lag persists

Several plausible drivers explain the slow translation of evidence into practice. They operate at the level of individual clinicians, professional groups, institutions, and the wider regulatory environment, and they are mutually reinforcing in ways that make incremental change difficult.

Historical Orthodoxy

The most fundamental driver is the weight of historical clinical orthodoxy. Decades of training, textbook narrative, case reports, and medico-legal precedent have framed the relationship as definitively unsafe, and the contraindication has been transmitted across generations of clinicians as a settled fact rather than an empirical claim. Reversing such an anchored belief requires more than the publication of new evidence; it requires sustained engagement through professional bodies, undergraduate and postgraduate curricula, mandatory continuing professional development, and structured exposure to the contemporary literature. Where the inertia is greatest, in primary care and in non-urological hospital practice, the new evidence has had the least visibility.

Medico-Legal Anxiety

Medico-legal anxiety is a further important driver. A recent analysis of TRT-related litigation in the United States identified PCa outcomes among the cited concerns in lawsuits against prescribers [33]. Although such cases have not consistently produced successful claims, the perception of medico-legal exposure remains a strong deterrent in everyday clinical decision-making. Clinicians considering TRT in a man with any history of PCa face an asymmetric regret calculus: the imagined consequences of a feared and unlikely recurrence on TRT loom larger in clinical imagination than the slow, less visible consequences of leaving symptomatic hypogonadism untreated. In the United Kingdom, where the General Medical Council’s good-practice framework expects clinicians to weigh benefits and harms transparently, the asymmetry is no less powerful. Clinicians may find it easier to decline therapy than to engage with the evidence and document a shared decision.

Uncertainty About On-Treatment Monitoring

Concern about on-treatment biochemical monitoring is perhaps the most clinically specific driver. Even where clinicians are willing in principle to consider TRT, the prospect of interpreting PSA fluctuations during testosterone supplementation creates a separate and powerful barrier. Small rises in PSA, which are clinically benign in the great majority of contexts, can prompt premature TRT discontinuation, additional biopsies, or escalation to active treatment. Without clear protocols for distinguishing benign androgen-driven PSA changes from genuine progression signals, on-treatment monitoring is experienced as an open-ended liability rather than a structured surveillance commitment. The empirical literature provides substantial reassurance; the consistent observation across cohorts is that PSA does not rise meaningfully during physiological-range TRT, with the modest exception of small expected increases reflecting normalisation of the hormonal milieu [10,18,21]. However, the reassurance has limited clinical traction unless individual clinicians have explicit, locally applicable protocols to fall back on.

Absence of Randomised Trial Data

The absence of randomised controlled trial data specifically in men with treated PCa is a structural problem that constrains guideline panels. Although the volume of observational data is now substantial, true randomisation in this population is ethically difficult, as it would require either offering placebo to symptomatic hypogonadal men with treated PCa or randomising them to active comparator interventions of uncertain equivalence. Funding for such a trial is also unlikely to materialise, given the off-label status of the prescription and the modest commercial incentive. Guideline panels that emphasise GRADE methodology and high-level evidence are therefore constrained from issuing strong recommendations on observational data alone, even when the direction and consistency of that data are reassuring. The threshold for changing a long-standing contraindication is appropriately high; the practical consequence is that the threshold may not be easily reached.

Multidisciplinary Fragmentation

Multidisciplinary fragmentation is a further driver, particularly pronounced in the NHS. TRT in PCa survivors sits at an unusual professional interface between urological oncology, andrology, sexual medicine, endocrinology, and primary care. In most healthcare systems, no single clinician routinely owns this conversation, and the patient’s symptoms tend to be raised opportunistically rather than systematically reviewed. Survivors discharged from oncological follow-up are often left to raise concerns about hypogonadal symptoms with general practitioners who, understandably, defer to specialist guidance that does not exist in clearly actionable form. The result is repeated cycles of deferred decisions and unaddressed symptoms. Where dedicated andrology services or men’s health clinics exist within an integrated cancer pathway, this fragmentation is partially mitigated, but such services remain unevenly distributed across NHS regions and largely absent in many smaller centres.

Training and Education

Training deficits compound the structural problem. TRT initiation in men with treated PCa is not a routine component of UK urology training and is not formally addressed within the current Joint Committee on Surgical Training curriculum for urology. Many registrars complete CCT without ever having initiated TRT in such a patient or observed a senior colleague do so. The hidden curriculum reinforces the historical contraindication; the explicit curriculum is silent. New consultants are then asked to make decisions in clinic for which their training has provided neither structured exposure nor explicit framework. The same is true at undergraduate and foundation level, where the topic is virtually absent. Without deliberate curricular change, the gap between evidence and practice will reproduce itself through each successive cohort of clinicians.

Public Discourse Around Testosterone

The polarised public conversation around testosterone has also not helped. The expansion of direct-to-consumer testosterone clinics, online prescribing channels, and intensified media attention to the overdiagnosis of “low testosterone” has framed TRT as a commercial enterprise as much as a clinical intervention. Legitimate concerns about over-prescription in otherwise healthy men have at times been conflated with the distinct clinical question of under-prescription in symptomatic survivors of treated PCa. The combined effect is a defensive clinical atmosphere in which both groups of patients are poorly served: the worried well receive testosterone they may not need, while symptomatic survivors are routinely denied a discussion that the contemporary evidence supports. Restoring the prescriber’s confidence to engage with the latter group requires distinguishing the two questions clearly in professional, educational, and patient-facing material.

A path forward

The gap between evidence and practice is not principally a problem of further evidence, although prospective registries and long-term follow-up data remain valuable. It is a problem of translation, and closing it requires deliberate action across guideline development, clinical service design, training, and patient-facing resources.

Guideline language is the most influential lever. The American Urological Association testosterone deficiency guideline is now eight years old and predates the most informative contemporary studies. A revised statement reflecting the data of 2024-2026, including the Memorial Sloan Kettering post-prostatectomy cohort, the SEER-Medicare active surveillance analysis, the TRAVERSE prostate safety substudy, the Garcia-Becerra meta-analysis, and the recent systematic and scoping reviews, would substantially shift the perceived basis of clinical decision-making. In the United Kingdom, the BSSM 2026 consensus offers a contemporary template that could be incorporated into National Institute for Health and Care Excellence guidance on hypogonadism and into British Association of Urological Surgeons (BAUS) practice statements on survivorship care. NHS commissioning of TRT, which is currently inconsistent across regions, would also benefit from explicit reference to the post-cancer survivor population in commissioning policy.

Standardised shared decision-making tools are a complementary need. Patients deserve structured, evidence-based conversations about TRT after PCa treatment. A useful framework should explicitly address the absence of an absolute contraindication in contemporary evidence; the relevance of disease characteristics, including risk group, modality of treatment, current PSA, and time since treatment; the off-label nature of the prescription; the surveillance commitment required, including the interpretation of expected PSA changes; and the patient’s own values regarding symptoms, function, and acceptable uncertainty. Patient-decision aids developed and validated for this conversation, ideally in collaboration with cancer charities such as Prostate Cancer UK and Macmillan, would support both consistency of practice and patient empowerment.

Multidisciplinary survivorship pathways are essential to operationalise the evidence at scale. A clinically significant proportion of PCa survivors would benefit from structured assessment of post-treatment hypogonadism, and this is best delivered through integrated survivorship clinics with input from urology, clinical oncology, sexual medicine, endocrinology, and primary care, rather than left to ad hoc post-discharge presentations. Pathway design should specify how and when hypogonadal symptoms are screened, who is responsible for biochemical confirmation, where the TRT decision is made, and how shared care with primary care is structured. NHS England’s PCa survivorship pathway, and equivalent frameworks in Scotland, Wales, and Northern Ireland, would each benefit from explicit reference to hypogonadism assessment and the option of TRT consideration.

Prospective international registries enrolling men receiving TRT after PCa treatment, with standardised surveillance protocols and long-term follow-up, would provide the granularity that no individual cohort can. Such registries should include diverse populations, particularly Black men, who are underrepresented in existing data despite their higher PCa incidence, and intermediate- and selected high-risk subgroups, in whom the current evidence base is thinnest. A UK component, embedded within BAUS or BSSM structures and linked to national cancer registry data, would be feasible at modest cost and would yield evidence directly applicable to NHS practice.

Training and education must be reformed in parallel. Urology curricula at registrar level should include explicit teaching on TRT in PCa survivors, ideally embedded within the JCST curriculum framework. Surgical training programmes should ensure that registrars encounter these decisions in supervised settings before becoming consultants who must make them independently. Continuing professional development for established consultants, primary care educators, and specialist nurses should reflect contemporary evidence. The European Association of Urology and BAUS annual congresses, FRCS revision courses, and Royal College training events all offer existing structures into which updated content could be incorporated without additional infrastructure.

Finally, patient information should be improved. Plain-language, evidence-based patient resources on TRT after PCa are sparse and frequently inaccurate, oscillating between reassurances from commercial TRT clinics and reflexive cautions from oncological resources. National-level resources from cancer charities and professional societies should fill this gap, providing balanced summaries of the evidence, an honest acknowledgement of the residual uncertainties, a clear description of what monitoring on TRT involves, and signposting to local services. An empowered patient population, equipped to raise the question and to engage with the surveillance commitment, is one of the most effective drivers of practice change.

## Conclusions

The contraindication to TRT in men with PCa rests on decades-old observations in a small number of patients with advanced metastatic disease. In more recent literature, the underlying biology has been substantially reframed, the empirical safety of TRT in carefully selected men has been established across multiple study designs and populations, and professional consensus has begun to formalise the contemporary clinical position. The historical absolute contraindication is increasingly difficult to justify. What remains is a practice gap. Closing it will require updated guideline language, structured shared decision-making, multidisciplinary survivorship pathways, prospective data collection, deliberate training reform, and improved patient information. It also requires recognising that the caution that shaped this area for decades has had real costs for patients. Clinical practice now needs to align more closely with contemporary evidence.
